# Complete Genome Sequencing of Field Isolates of Peste des Petits Ruminants Virus from Tanzania Revealed a High Nucleotide Identity with Lineage III PPR Viruses

**DOI:** 10.3390/ani11102976

**Published:** 2021-10-15

**Authors:** Edson Kinimi, Mana Mahapatra, Tebogo Kgotlele, Mariam R. Makange, Chandana Tennakoon, Felix Njeumi, Steven Odongo, Serge Muyldermans, Richard Kock, Satya Parida, Mark Rweyemamu, Gerald Misinzo

**Affiliations:** 1 SACIDS Africa Centre of Excellence for Infectious Diseases, SACIDS Foundation for One Health, Sokoine University of Agriculture, P.O. Box 3297, Morogoro 67125, Tanzania; satya.parida@fao.org (S.P.); mark.rweyemamu@sacids.org (M.R.); 2 Department of Veterinary Physiology, Biochemistry and Pharmacology, College of Veterinary Medicine and Biomedical Sciences, Sokoine University of Agriculture, P.O. Box 3017, Morogoro 67125, Tanzania; 3 Department of Veterinary Microbiology, Parasitology and Biotechnology, College of Veterinary Medicine and Biomedical Sciences, Sokoine University of Agriculture, P.O. Box 3019, Morogoro 67125, Tanzania; tkgotlele@gmail.com (T.K.); mirrichy@gmail.com (M.R.M.); 4 The Pirbright Institute, Ash Road, Pirbright, Woking GU24 0NF, UK; mana.mahapatra@pirbright.ac.uk (M.M.); chandana.tennakoon@pirbright.ac.uk (C.T.); 5 Food and Agriculture Organization of the United Nations (FAO), Viale delle Terme di Caracalla, 00153 Rome, Italy; Felix.Njeumi@fao.org; 6 Department of Biotechnical and Diagnostic Sciences, College of Veterinary Medicine, Animal Resources and Biosecurity (COVAB), Makerere University, Kampala P.O. Box 7062, Uganda; opodongo@yahoo.co.uk; 7 Laboratory of Cellular and Molecular Immunology, Vrije Universiteit Brussel, Pleinlaan 2, 1050 Brussels, Belgium; Serge.Muyldermans@vub.be; 8 The Royal Veterinary College, University of London, Hawkshead Lane, North Mymms, Hertfordshire, Hatfield AL9 7TA, UK; Rkock@rvc.ac.uk

**Keywords:** peste des petits ruminants virus, PPR, Oxford nanopore MinION, diagnosis, complete genome, sequencing, Tanzania

## Abstract

**Simple Summary:**

Peste des petits ruminants virus (PPRV) causes a highly devastating disease, peste des petits ruminants (PPR), in sheep and goats, which is targeted for global control and eradication. However, in many developing countries, access to expensive sequencing technologies is limited and is compounded by difficulties in transporting clinical samples across international borders. Oxford nanopore MinION is a relatively cheap sequencing technology using portable devices that require minimal supporting laboratory infrastructure or technical expertise for sample preparation and rapid sequencing. In this study, Oxford nanopore MinION sequencing was carried out to generate complete genomes of PPRV from archived PPRV-positive samples collected from PPR outbreaks in goats in Ngorongoro and Momba districts in Tanzania during 2016 and 2018, respectively. Complete genomes of PPRV of 15,948 nucleotides long were generated within four hours of sequencing. The phylogenetic analysis of the complete genomes revealed a high nucleotide identity (96.19–99.24%) with lineage III PPR viruses currently circulating in East Africa, indicating a common origin. The Oxford nanopore MinION sequencer can be deployed to overcome diagnostic and surveillance challenges in developing countries in the PPR Global Control and Eradication program. However, the coverage depth was uneven across the genome and amplicon dropout was observed between the matrix (M) and fusion (F) genes. Thus, larger field studies are needed to allow the collection of sufficient data to assess the robustness of nanopore sequencing technology.

**Abstract:**

Peste des petits ruminants virus (PPRV) causes a highly devastating disease of sheep and goats that threatens food security, small ruminant production and susceptible endangered wild ruminants. With policy directed towards achieving global PPR eradication, the establishment of cost-effective genomic surveillance tools is critical where PPR is endemic. Genomic data can provide sufficient in-depth information to identify the pockets of endemicity responsible for PPRV persistence and viral evolution, and direct an appropriate vaccination response. Yet, access to the required sequencing technology is low in resource-limited settings and is compounded by the difficulty of transporting clinical samples from wildlife across international borders due to the Convention on International Trade in Endangered Species (CITES) of Wild Fauna and Flora, and Nagoya Protocol regulations. Oxford nanopore MinION sequencing technology has recently demonstrated an extraordinary performance in the sequencing of PPRV due to its rapidity, utility in endemic countries and comparatively low cost per sample when compared to other whole-genome (WGS) sequencing platforms. In the present study, Oxford nanopore MinION sequencing was utilised to generate complete genomes of PPRV isolates collected from infected goats in Ngorongoro and Momba districts in the northern and southern highlands of Tanzania during 2016 and 2018, respectively. The tiling multiplex polymerase chain reaction (PCR) was carried out with twenty-five pairs of long-read primers. The resulting PCR amplicons were used for nanopore library preparation and sequencing. The analysis of output data was complete genomes of PPRV, produced within four hours of sequencing (accession numbers: MW960272 and MZ322753). Phylogenetic analysis of the complete genomes revealed a high nucleotide identity, between 96.19 and 99.24% with lineage III PPRV currently circulating in East Africa, indicating a common origin. The Oxford nanopore MinION sequencer can be deployed to overcome diagnostic and surveillance challenges in the PPR Global Control and Eradication program. However, the coverage depth was uneven across the genome and amplicon dropout was observed mainly in the GC-rich region between the matrix (M) and fusion (F) genes of PPRV. Thus, larger field studies are needed to allow the collection of sufficient data to assess the robustness of nanopore sequencing technology.

## 1. Introduction

Peste des petits ruminants (PPR) is a highly contagious viral disease of wild and domestic small ruminants caused by peste des petits ruminants virus (PPRV), a pathogen targeted for global control and eradication by 2030 [1]. PPR has been reported at an increased rate from its historical distribution across Africa and Asia into new areas where it has not been detected previously [2,3] since the global eradication of rinderpest. Owing to its spread and expansion beyond its known geographical boundaries, PPR causes significant economic losses between USD 1.2 and 1.7 billion globally per year, due to decreased production and animal death, as well as the cost required to overcome the disease [4]. Approximately one third of the economic losses occur in Africa and a quarter in South Asia, and the rest in East Asia, the Middle East and West Eurasia including Turkey, with costs being incurred predominantly by subsistence farmers [5,6]. However, an investment of USD 7.1 billion could be recovered within five years of a successful global eradication, with a 33.8 benefit:cost ratio that makes PPR eradication economically feasible [4]. Unfortunately, the delay of the response following rinderpest eradication and following the identification of PPR spread across disease-free zones and susceptible animal populations such as *camelidae, suidae,* and *bovinae*, has increased the likely eradication cost [1]. For instance, the financial losses associated with an outbreak of PPR in critically endangered species of saiga antelope (*Saiga tatarica mongolica*) in Mongolia were estimated at USD 7.27 million [1]. In order to avoid unprecedented financial losses in both wild and domestic small ruminants, an intensive, mass vaccination programme is required to reach and maintain high levels of herd immunity in sheep and goats [7]. It would be more efficient to target vaccination to identified pockets of endemicity responsible for PPRV persistence and create high levels of vaccination immunity in these defined populations, as was successfully carried out in the last phase of rinderpest eradication [7,8]. The identification of pockets of endemicity responsible for PPRV persistence requires an active surveillance programme in susceptible animal populations with rapid and cost-effective diagnostic tools.

The development of the necessary tools for PPR control, including vaccines, diagnostics and therapeutics, greatly depends on in-depth genomic information on the virus [9]. Unfortunately, few complete genomes of PPRV exist, and very few from East Africa, despite the existence of lineage II, III and IV PPRV [10]. The publicly available PPRV sequences from Tanzania were partial sequences, based on nucleoprotein sequencing, to study the phylogeny of PPRV isolates (Table 1). This restricts the ability to define important changes in the genome outside of pre-defined target genetic markers. Nevertheless, genetic changes could be important in viral evolution studies and in the development of novel diagnostic and therapeutic tools for PPR [9,11]. Interestingly, Oxford nanopore MinION sequencing is a rapid and relatively cheap technology that uses portable devices that require minimal supporting laboratory infrastructure or technical expertise for sample preparation [12]. The relatively low abundance of viral nucleic acids compared to that of host nucleic acids in clinical samples often necessitates the analysis of a substantial amount of sequence data, reflected in analysis times and associated costs [13]. In this study, the ARTIC method, based on tiling multiplex PCRs that were previously used to enrich PCR amplicons for Zika virus, Ebola virus and severe acute respiratory syndrome coronavirus-2 (SARS-CoV-2), was adopted to generate the complete genomes of field isolates of PPR [13,14]. Using this advanced approach, by assessing the long overlaps among multiplex amplicons, the accurately assembled and complete viral genome can be obtained, which can facilitate the rapid genomic surveillance of PPRV for better understanding its pathogenicity, evolution and transmission. Although a protocol for Oxford nanopore sequencing of PPRV has been established, adoption of the technology has been limited due to concerns around accuracy and high error rates associated with homopolymer lengths [15]. The nanopore device exhibits lower read-level sequencing accuracy than its short-read platform counterparts [16]. Moreover, the longest untranslated intergenic region between *M* and *F* genes, with about 66–72% GC content, impedes full-genome sequencing with next-generation sequencing technologies such as Illumina and Oxford nanopore MinION [9,17]. This region is an extremely difficult PCR target to sequence due to repetitive sequences and secondary DNA structure formation. In order to circumvent this deficiency, a method was successfully developed to amplify the GC-rich fragments (F7, F8 and F9) by redesigning long-read primers targeting the GC-rich region, prior to the full-genome sequencing in another study (manuscript in preparation). 

The etiological agent PPRV belongs to the genus Morbillivirus of the family *Paramyxoviridae* [18]. The PPRV genome is linear, non-segmented, negative-sense, single-stranded RNA, which is 15,948 nucleotides long [19]. Its genome length complies with hexamer length and the “rule of six,” in which the total number of nucleotides must be a multiple of six for the virus to replicate efficiently in infected cells [19]. However, a longer variant of PPRV of 15,954 nucleotides long has been reported in China [20]. The tolerance for variation is particularly constrained in the genomic termini, as they contain essential elements for replication, such as the signal for encapsidation and promoters for genome and antigenome replication [20,21]. Generally, this genome comprises six genes in order of 3′-N, P, M, F, H, L-5′, with each gene coding for a distinct structural protein, the exception being the phosphoprotein (P) gene [9,19] which also codes for additional non-structural proteins. These non-structural proteins, designated C and V, are generated through alternative start codons (leaky scanning) and RNA editing, respectively [22]. The encoded proteins bear the acronym of the respective gene of origin; the nucleoprotein (N), the phosphoprotein (P), the matrix protein (M), the fusion protein (F), the haemagglutinin protein (H) and the RNA-dependent RNA polymerase also known as large protein (L). The N protein is abundant in PPRV-infected cells because the *N* gene is located near the 3′ proximal genomic promoter, and hence it is the most transcribed gene [23]. Given its abundance and antigenic stability, the N protein is frequently used in PPR diagnostic development and is the most appropriate gene for molecular characterization of closely related isolates [24]. Based on the partial molecular genetic characterization of N and fusion F protein genes, PPRV has been grouped into four distinct lineages (I, II, III and IV) that exist as a single serotype [25,26,27]. 

The current geographical distribution of PPRV lineages shows lineages I, II and III as restricted primarily to the African continent [10], whilst lineage IV is found throughout Asia and the Middle East, although early detection includes Central Africa as well as contemporary detections in North and sub-Saharan Africa [2,10]. In 2008, lineage IV PPRV was reported to have caused a devastating epidemic of PPR in Morocco [28]. Phylogeographic analysis suggests that lineage IV PPRV has spread from Eastern Africa, most likely from the Sudan 2000 outbreak, into Northern Africa, resulting in the 2008 Moroccan outbreak [29]. Later in 2009, lineage IV was also detected in several countries in sub-Saharan Africa [25,30,31,32], where there was no clear-cut association with trade of movement from either Morocco, the Middle East or South Asia. In the 1990s, Tanzania was free from PPR based on comprehensive serological investigations in goats and sheep [33]. In 2008, PPR was serologically confirmed in northern Tanzania for the first time [34]. However, a retrospective study that was conducted on archived samples collected from Ngorongoro district between 1998 and 2004 found antibodies against PPRV already present in samples collected in 2004, indicating that PPR might have been present in Tanzania before it was officially confirmed in 2008 [35]. Molecular confirmation of PPRV was carried out at a much later time (2010) than would have been expected for the correct identification of outbreaks (Table 1). 

Since the 1980s, the diagnosis of PPR has constantly been improved through advances in material sciences, genomics, bioinformatics, biotechnology, nanotechnologies, microfluidics and the miniaturization of electronic devices [36]. The first partial nucleotide sequence of PPRV was generated from the cloned N gene of PPRV/N/75/1 vaccine strain in 1994, which was followed by the sequencing of the F protein gene [37,38]. The sequencing of the *N* and *F* genes was pivotal in the development of important molecular diagnostic tools for PPR detection and confirmation [26,39]. More importantly, in 2005, a complete genome of PPRV was publicly available for the first time using Sanger standard methods [19]. Sanger dideoxynucleotide cycle sequencing has been a standard method for the sequencing of PPRV and confirmation of sequences that are difficult to generate with other methods [10,19,40]. With recent advances in sequencing technologies, the complete genomes of PPRV isolates are now being generated using next-generation sequencing technologies, mainly Illumina, and recently Oxford nanopore MinION [17]. The Oxford nanopore MinION sequencing technology has been proven powerful in the genetic characterization of infectious disease agents, including PPRV [13]. The establishment and deployment of nanopore sequencing of PPRV provides an opportunity for the molecular epidemiological surveillance of PPR in resource-limited settings and challenging geographical landscapes, due to its rapidity, improved accuracy and relatively low sequencing cost per sample [12]. This sequencing tool may play a significant role in controlling PPR outbreaks, enabling the detection of cryptic foci, inadequate vaccine deployment and other challenges in the midst of an eradication campaign. Moreover, Oxford nanopore has recently shown a capability for sequencing a full or nearly full genome of PPRV in a single sequencing run within 4 h of sequencing. This provides an opportunity for genomic surveillance of PPRV in real time, necessary for the early implementation of control measures in low-income and in resource-constrained countries, where PPR is endemic. 

The present study was undertaken to generate complete genomes of PPRV isolates collected from goat in the Ngorongoro and Momba districts of Tanzania in 2016 and 2018, respectively, using the Oxford nanopore MinION sequencer. The use of the Oxford nanopore MinION sequencer permits sequencing of PPRV in resource-limited settings and in addition makes the production of a complete genome possible within a day. The availability of the full genome of PPRV provides important insight in viral evolution, transmission routes and the implementation of appropriate control measures.

## 2. Materials and Methods

### 2.1. Sample Source Description and Storage

**Figure 1:**
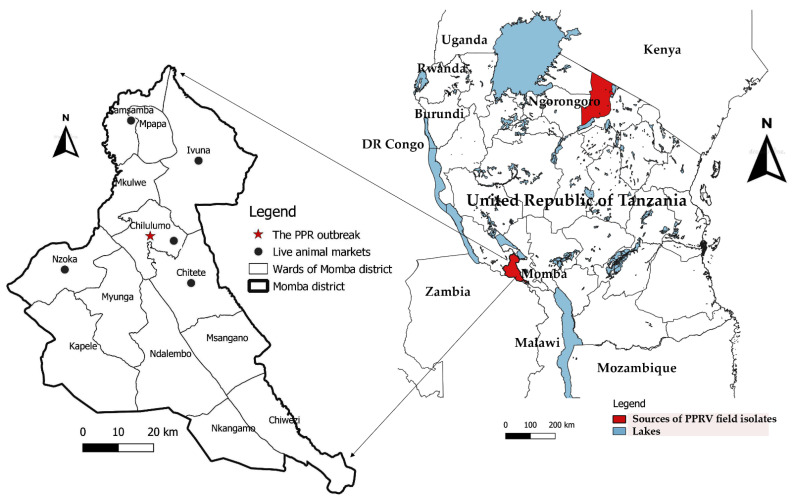
A map of Tanzania showing sources of PPRV field isolates used in this study. The archived PPRV-positive samples analysed in this study were collected from goats in Chilulumo ward in Momba district and in Loliondo area in Ngorongoro district, marked in red.

**Figure 2:**
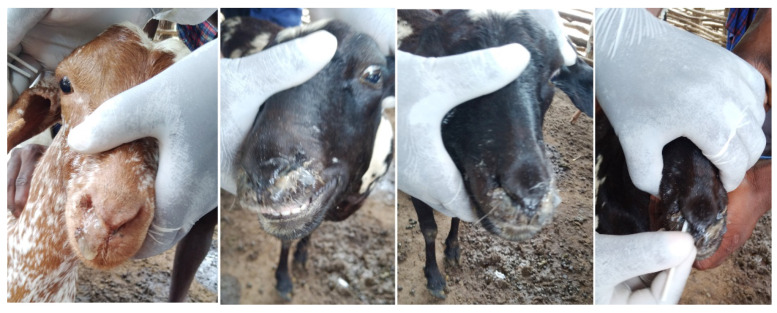
Nasally discharging goats suggestive of peste des petits ruminants virus (PPRV) infection, during a peste des petits ruminants (PPR) sero-survey at Chilulumo ward in the Momba district, in 2018. The nasal swab samples were collected and tested for the presence of PPRV infection. The PPR-positive samples were stored at −80 °C for further research.

The nasal swab samples used in this study were collected from goats in Chilulumo ward in Momba district and the Loliondo area of Ngorongoro district in 2018 and 2016, respectively. Momba district is located in the north-western part of the Songwe region of the Southern highlands of Tanzania (Figure 1). The district borders the Rukwa region and Zambia to the west, with Mbozi district to the east, Chunya district to the north and Ileje district to the south, whilst the Ngorongoro district is located in the northern part of the country in the Arusha Region. The samples collected from the Loliondo area of Ngorongoro district in 2016 were previously described and tested by Kgotlele et al. [47]. However, Momba samples were collected during PPR sero-survey in September, 2018. The key clinical signs investigated in 13 goats suspected of PPRV infection include; fever, nasal and ocular discharges, diarrhoea and laboured breathing (Figure 2). The rectal temperature of clinically sick goats ranged between 40 and 41.5 °C, with an average temperature of 41 °C. It was noted that all sick goats (*n* = 51) recruited for study were an indigenous breed aged between 1 and 2 years. The study recorded 10 deaths with case fatality rates of 19.6% and 100% morbidity. The nasal swabs from live goats were collected in universal viral transport medium (BD Biosciences, Maryland, USA) followed by flicking to dislodge cells from the swabs, and were stored at −80 °C until RNA extraction was undertaken.

### 2.2. Sample Selection

Seventy-three samples that were previously screened for PPR from different outbreaks in Tanzania were used in this study. When the samples were re-tested by conventional reverse transcription polymerase chain reaction (RT-PCR) prior to full-genome sequencing, most were negative for PPRV, indicating RNA degradation due to temperature fluctuations in our ultralow temperature freezers (freeze and thaw) as a result of frequent power cuts [26]. For example, all 36 samples collected during a PPR outbreak in Tandahimba in 2011 were negative. A total of 7 out of 24 samples collected during a PPR outbreak in Ngorongoro were positive. Out of these 7 PPRV-positive samples, only one had a strong visible band on agarose gel electrophoresis after RT-PCR. Similarly, only 1 out of 13 samples collected during a PPR outbreak in Momba in 2018 produced a strong visible band on agarose gel electrophoresis after RT-PCR. Thus, in total we had 8 PPRV-positive samples with only 2 strongly positive samples that yielded full PPRV genomes after next-generation sequencing. The remaining samples had very low PPRV genome coverage. 

### 2.3. RNA Extraction, cDNA Synthesis and PCR Amplification

Total RNA was extracted using the QIAamp Mini kit (Qiagen, Hilden, Germany) according to the manufacturer’s instructions. The cDNA synthesis was carried out using the Superscript IV First-Strand Synthesis System (Invitrogen, Paisley, UK) using 11 µL of RNA, according to the full wet lab protocol [17]. Polymerase chain reaction (PCR) nucleotide amplification reactions were performed using the Q5 Hot Start High Fidelity Polymerase (New England BioLabs, UK). Twenty two pairs of multiplex primers of 800 bp with an overlap of 100 bp were used as previously described by Torsson et al. [17], plus three newly designed pairs of primers targeting the long guanine cytosine untranslated region (GC-rich) of PPRV (Table 2). Briefly, two separate PCR reactions were carried out for each PPRV-positive sample. Two pools of primers were made; ‘pool 1′ contained eleven primers that generated the odd-numbered tiled amplicons, while ‘pool 2′ contained eleven primers that generated the even-numbered tiled amplicons for the 800 bp set. The three primers targeting the GC-rich fragments (F7, F8 and F9) were used separately in each PCR tube. The PCR reactions were performed using a nexus gradient master thermocycler (Eppendorf AG Hamburg, Germany). The resulting PCR amplicons were pooled and then purified using AMPure XP magnetic beads (Beckman Coulter, Redwood, USA) with a 1.8x bead ratio and quantified using Qubit 1.0 Fluorometer dsDNA HS assay (Thermo Fisher Scientific, Waltham, USA). The PCR amplicons were run on a 1% agarose gel and visualized using a Gel Doc^TM^ EZ Imager agarose gel imaging system (Bio-Rad, Hercules, CA, USA). The detailed methods for reverse transcription, tiled multiplex PCR and library ligation were followed according to the full wet laboratory protocol available (doi.org/10.17504/protocols.io.pnxdmfn) since April 2021.

### 2.4. Nanopore Library Preparation and Sequencing

Sequencing libraries were prepared using the SQK-LSK109 ligation sequencing kit and EXP-NBD104 native barcode expansion (Oxford Nanopore Technologies, UK) following the nanopore sequencing protocol [17]. The concentration of 0.12 pmol PCR products was diluted in 25 µL of nuclease-free water. This generated 60 ng of the PCR product in 25 µL water for our amplicons (800 bp) (https://nebiocalculator.neb.com/ accessed on 11 November 2020). The final concentration of the DNA library was 42 fmol. Briefly, the purified PCR amplicons were repaired and A-tailed using the NEB Next Ultra II End Repair/dA-Tailing module (New England BioLabs, Ipswich, MA, USA). Native barcodes and adaptors were ligated to end-repaired PCR amplicons using Blunt/TA Ligase Master Mix (New England BioLabs, Ipswich, MA, USA), to generate the nanopore library. The library was then sequenced on a MinION Flow cell for 4 h. The nanopore sequencing raw read datasets were generated in standard fast5 format. The nanopore raw reads were basecalled and demultiplexed to generate fastq files using GUPPY software built in MinIT. 

### 2.5. Nanopore Dataset Analysis

The composition and quality of reads were assessed using nanoplot and qScore, and the reads below a qScore of 7 were removed by the EPI2ME software version 2019.7.9, (as per ONT pass/fail threshold) before downstream analysis (PycoQC (https://usegalaxy.org, accessed on 5 January 2021). Additional demultiplexing and adaptor removal were performed using porechop in NanoGalaxy platform [49,50]. The trimmed nanopore reads were checked for purity using Q-score in the nanoplot [49,51]. The reads were aligned to the PPRV reference genomes (RefSeq accession number: KM463083) using minimap2 version 2.17 [52]. The resulting binary alignment map (BAM) file was sorted and converted into an indexed BAM file for additional processing with samtools version 1.9 [53]. Following this, a consensus sequence was created by obtaining the majority vote for the bases from the pileup of the BAM file. The Katuali (https://github.com/nanoporetech/katuali, accessed on 7 January 2021) pipeline was used to assemble the genome. The reads were assembled with Canu version 2.0 [54] and polished with Racon and Medaka (https://github.com/nanoporetech/medaka, accessed on 7 January 2021) [55]. For the Momba/Tanzania/2018 PPRV field isolate, the missing 5′ region (from 15890 bp onwards) in the nanopore assembly was completed using the matching region from the consensus. When the final assembly was compared with KM463083, 11 indels were found. We did not observe any clustering of the indels. We manually removed the indels where the frequency of the reads supporting an indel was below 40% and the frequency of the reads that did not support the indel exceeded 60%. The browser extensible data (BED) files were created, representing the coverage of the sequence reads against the reference genome, and the results were visualized using integrative genomics viewer (IGV). Finally, the consensus sequences were annotated using genome annotation transfer utility [56] and whole-genome comparison was performed using the basic local alignment search tool (NCBI BLAST version 2.12.0, Rockville Pike, MD, USA). With comparable sequences from GenBank, a phylogenetic tree was constructed using the Maximum likelihood method, and the Kimura 2-parameter model with a bootstrap frequency of 1000 replicates, as implemented in MEGA X software [57]. 

### 2.6. Temporal Phylogenetics

To identify the nearest common ancestor and hence likely dates of divergence, the Tanzania/Momba/2018 and Tanzania/Ngorongoro/2016 (Accession Numbers: (MZ322753 and MW960272) sequences were compared using the coalescent-based Bayesian Markov chain Monte Carlo (MCMC) approach to selected complete genomes of PPRV available in GenBank (*n* = 38) [58]. The gamma distribution and general time-reversible nucleotide substitution model for rate variation and the proportion of invariant sites were selected on the basis of Akaike information criterion scores. Bayesian time-scaled phylogenetic analysis molecular evolutionary rate and divergence times were estimated. A Bayesian maximum clade credibility (MCC) phylogenetic tree was constructed by using Bayesian Markov chain Monte Carlo (MCMC) analysis and Bayesian evolutionary analysis sampling trees (BEAST), and the Tree Annotator software package v1.10.4 (Auckland, New Zealand). For the sequence dataset, the best-fit nucleotide substitution model was determined on the basis of Akaike information criterion scores using JModel Test software v2.1.4 (Boston, MA, USA), as previously described [59]. An input file for BEAST analysis was obtained by using Bayesian evolutionary analysis utility software, BEAUti v1.10.4 (Auckland, New Zealand) in which sequences were tip dated according to the year of collection. The relaxed molecular clock with coalescent exponential growth was the most appropriate model for this analysis, as previously reported [29,59]. The Bayesian analyses were run for 50,000,000 iterations sampled every 5000 in duplicate; duplicate runs were combined for final analysis with effective sample size (ESS > 200) and were assessed for their proper mixing, convergence and consistency by Tracer v1.7.2 with 10% burn in. The two individual runs were combined by using LogCombiner v1.10.4 in the BEAST software package (Auckland, New Zealand). The nucleotide substitution rate (substitutions/site/year) and the time to the most recent common ancestor (TMRCA) (year) values were obtained from Tracer v1.7.2. The posterior tree distributions were summarized by using TreeAnnotator v1.10.4 and exclusion of the first 10% of the trees as burn in. Phylogenetic MCC tree with median node heights were visualized in FigTree software v1.4.2 (Auckland, New Zealand). The phylogenetic MCC tree in Newick file format together with metadata file with accession number and country of origin were uploaded to PastML for ancestral state reconstruction (https://pastml.pasteur.fr/, accessed on 2 May 2021).

## 3. Results

### 3.1. Nucleotide Amplification

**Figure 3:**
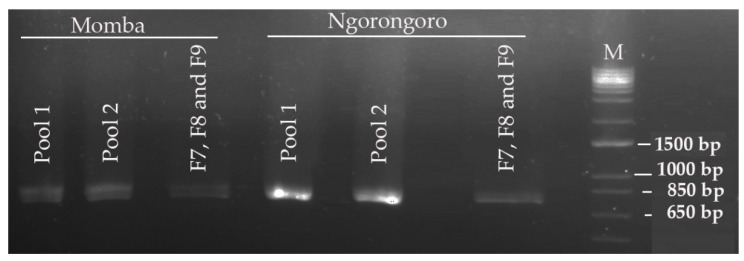
Gel electrophoresis of tiling multiplex polymerase chain reaction (PCR) amplification products of peste des petits ruminants virus isolates from Momba and Ngorongoro districts in Tanzania. The PCR amplicons indicate an expected band size of around 800 bp in accordance with primer targets.

Out of eight archived PPRV-positive samples screened for PPR, only two samples had strong bands on agarose gel electrophoresis using gene-specific primers (NP3/NP4) targeting the nucleocapsid protein gene. The two positive samples were collected from goats in Ngorongoro and Momba districts in the northern and southern highland of Tanzania. The cDNA obtained from these positive samples were used subsequently in tiling multiplex PCR. Analysis of the PCR amplicons on agarose gel electrophoresis exhibited strong PCR bands of expected size (~800 bp), confirming successful amplification of the fragments (Figure 3). However, relatively faint bands were observed in the PCR fragments that targeted the GC-rich region of PPRV.

### 3.2. Long-Read Nanopore Sequencing of PPRV Field Isolates

**Figure 4:**
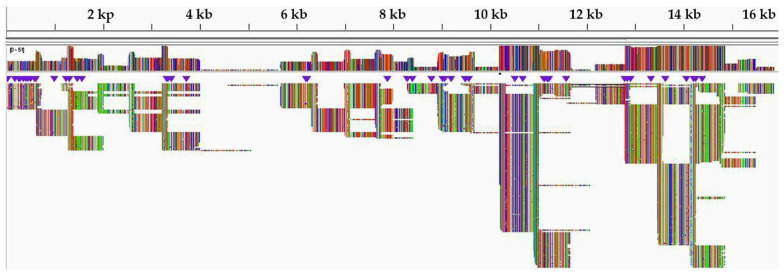
Integrative genomic viewer (IGV) of peste des petits ruminants virus (PPRV) sequence reads against reference genome (KM463083).

Sequencing of PCR amplicons produced over 1.5 million raw reads from each sample (Table 3). The read coverage from this isolate was much higher, up to 8000 reads per position, with low reads (34–163 per position) in the GC-rich region between the 4 and 6 kb genome positions (Figure 4). Similarly, low reads were observed in the leader and trailer regions, as low as 189 and 13 per position, respectively. In the region where a missing region was constructed using the consensus for the Momba sequence, the average read coverage was 84.4.

### 3.3. Annotation of Peste des Petits Ruminants Virus Isolate

**Figure 5:**
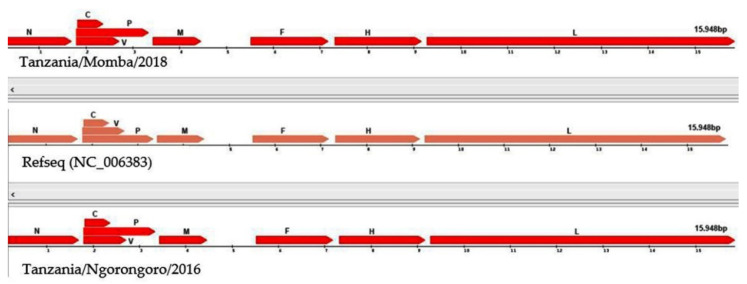
Graphical view of peste des petits ruminants virus (PPRV) open-reading frames (ORFs) in comparison with reference sequence (NC_006383), using genome annotation transfer utility.

The nanopore sequencing of PPRV field isolates generated full-length genomes with 15,948 nucleotides. The graphical view of the PPRV open-reading frames (ORFs) in comparison with the reference sequence showed that the PPRV genomes encoded six structural proteins with transcription units for the N, P, M, F, H and L proteins, and two non-structural proteins C and V transcribed from the *P* gene, using the genome annotation transfer utility (Figure 5). The Tanzania/Ngorongoro/2016 PPRV isolate showed the highest protein identity with lineage III isolate, KN/2011(KM463083.1), N (99%), P (99.8%), V (99.33%), C (100%) M (100%), F (98%), H (100%) and L (99.7%). Equally, high protein identity was observed with the Tanzania/Momba/2018 PPRV isolate, N (99.6%), P (98.4%), V (95.97%), C (97.7%) M (100%), F (99.5%), H (99.3%) and L (99.4%). Moreover, the PPRV isolates, Tanzania/2016 and 2018, contained 1080 nucleotides of untranslated region (UTR) at the genome position between nucleotides 4446 and 5526, with 66.9 and 69.4% GC contents, respectively, between the M and F open-reading frames.

### 3.4. Phylogenetic Analysis of Peste des Petits Ruminants Virus

**Figure 6:**
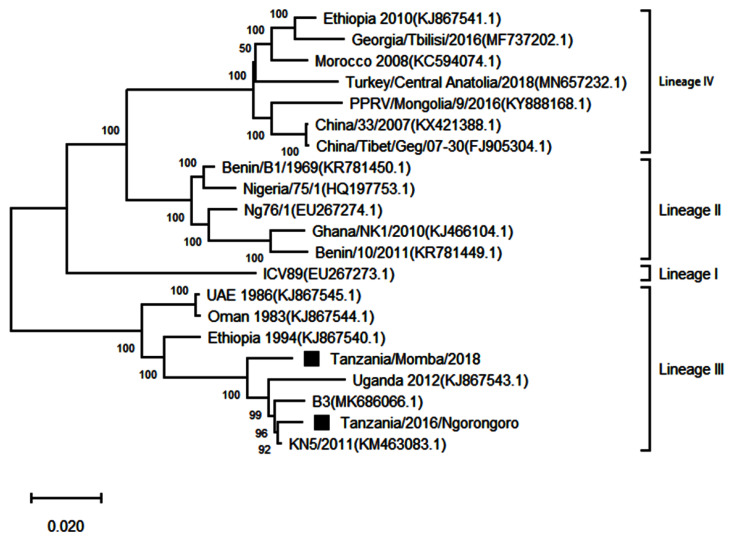
Maximum likelihood phylogenetic tree obtained after multiple sequence alignment of complete genomes of peste des petits ruminants virus strains from East Africa and selected strains from other parts of Africa and Asia. The viruses described in this study are indicated by black squares and the scale bar indicates nucleotide substitution per site, while the node values show percentage of bootstrap support. The analysis involved 21 nucleotide sequences with 15,962 positions in the final dataset.

Two complete genome sequences obtained from this study were submitted to GenBank and assigned accession numbers MW960272 and MZ322753. Another 19 full genomes representing all the four lineages were retrieved from GenBank for further analysis, making it a total of 21 sequences. The sequences showed good conformity with the PPRV complete genome sequences available on GenBank (Table 4). A phylogenetic analysis of the sequences showed that the PPRV sequences obtained from Loliondo in Ngorongoro and Chilulumo in Momba districts clustered into lineage III of PPRV (Figure 6). The comparison of their full genomes with those of other PPRV strains revealed the highest nucleotide identity (96.19 to 99.24%) with the PPRV isolate KN5/2011 (KM463083.1) from Kenya; B3 isolate from Burundi (MK686066.1) and Ugandan isolate (KJ867543.1).

### 3.5. Temporal–Spatial Spread of Peste des Petits Ruminants Virus

**Figure 7:**
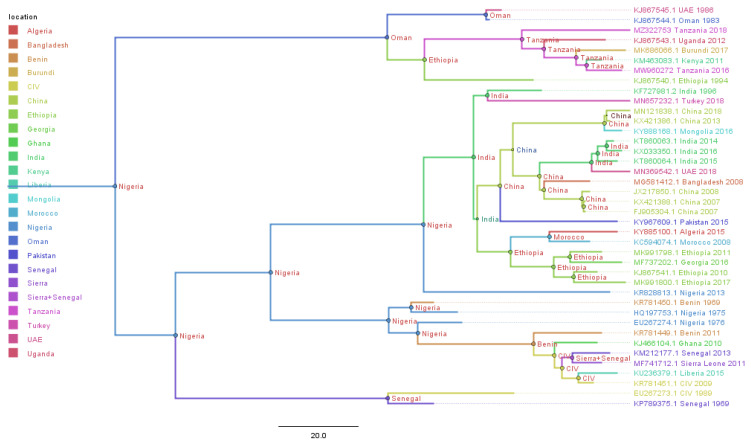
Maximum clade credibility (MCC) trees constructed for the phylogeographical reconstruction of peste des petits ruminants virus PPRV using complete genomes. Posterior probability values are indicated by the size of the node and posterior probability distribution, indicated as a location at the side of each node. Branches are coloured according to the most likely location at the preceding node in the tree. The year of the samples for which PPR viruses were sequenced and GenBank accession numbers are given against each sequence. A phylogeographic method estimated the probability of the root location of an ancestral PPRV and individual lineages as being Nigeria for PPRV, as previously reported by Muniraju et al., 2014.

**Figure 8:**
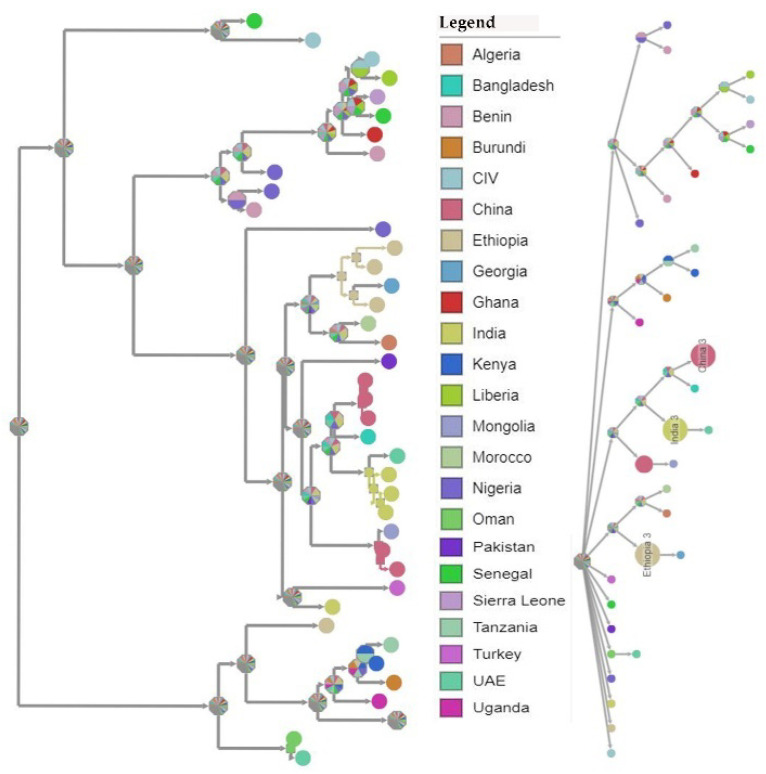
Ancestral rooted phylogenetic map of the selected complete genomes of peste des petits ruminants (PPR) viruses with annotated tips, using maximum likelihood (https://pastml.pasteur.fr/, accessed on 3 May 2021).

**Figure 9:**
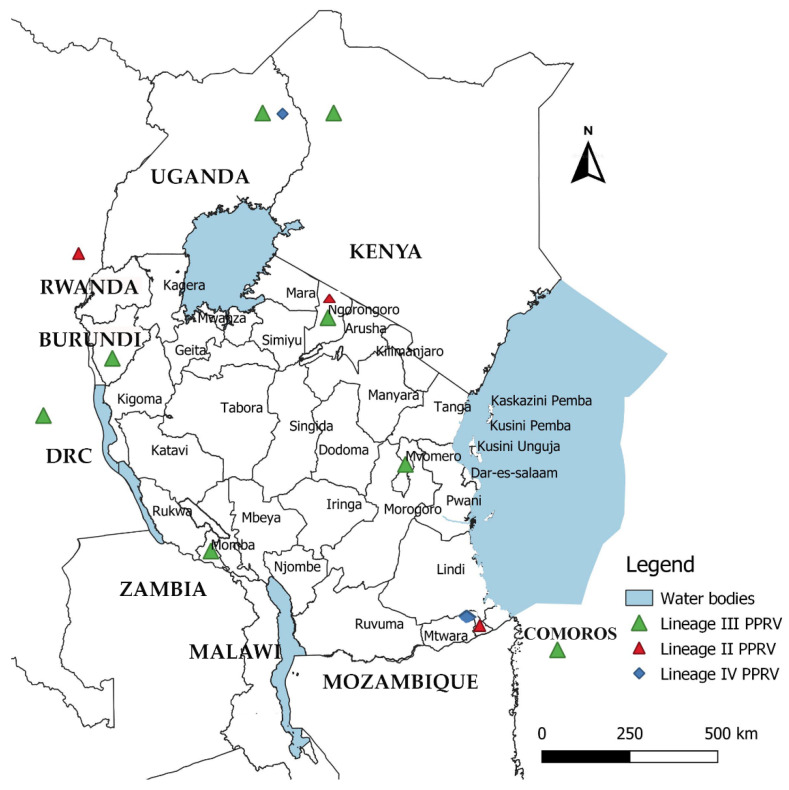
Distribution and molecular characterization of peste des petits ruminants virus in Tanzania and neighbouring countries (Kenya, Uganda, DR Congo, Burundi and Comoros). The colour of triangles and diamonds indicate the viral lineages reported in these countries: lineage III, light green; lineage II, red; and lineage IV, blue. This map was created using QGIS software version 3.16.8 downloaded from https://qgis.org/en/site/, accessed on 12 June 2021.

A Bayesian time-scaled MCC tree using complete PPRV genomes was constructed (*n* = 40), alongside Tanzania/Momba/2018 and Tanzania/Ngorongoro/2016 (Figure 7). In estimation of the route of entry of lineage PPRV III into East Africa, we visualized the summarized results of the Bayesian phylogeographic analysis of the complete genome (Figure 8). Analysis of the posterior probabilities suggests a strong historical and geographic connection between the Tanzanian isolates and PPR viruses isolated in East Africa (Figure 9). These analyses show a very strong likelihood >72% that the lineage III viruses currently circulating in East Africa are closely related to and, in the absence of further material, likely originated from the Ethiopia 1994 outbreaks (Figure 7).

## 4. Discussion

Peste des petits ruminants is an economically important transboundary animal disease of sheep and goats, which is targeted for global control and eradication, as a joint initiative of the Food and Agriculture Organisation of the United Nations (FAO) and World Organisation for Animal Health (OIE). Molecular epidemiological surveillance using in-depth genomic data has the potential to clarify the roles of a wide host range in PPRV circulation, direction of transmission at domestic–wildlife interfaces and how viral evolution may alter host range and virulence [73]. However, in many developing countries, access to the required sequencing technology is limited, compounded by the difficulty in transporting wildlife clinical samples across international borders due to the Convention on International Trade in Endangered Species of Wild Fauna and Flora, and Nagoya Protocol regulations [3]. The Oxford nanopore sequencing devices are portable, cheap, require minimal supporting laboratory infrastructure or technical expertise for sample preparation and can be used to perform rapid sequencing analysis with flexible scalability. Thus, the present study was carried out to generate complete genomes of PPRV field isolates using the Oxford nanopore MinION sequencer. With nanopore sequencing, two complete genomes of PPRV were generated within four hours. The resulting sequences had the same length as all of the other PPRV genomes sequenced to date and were in agreement with the ‘‘rule of six’’ for paramyxoviruses [19,20]. The organization of the genome was the same as those described previously, with transcription units for the N, P(C/V), M, F, H and L proteins [9]. The complete genomes generated with this technology comply with molecular epidemiology sequencing standards, as being “Coding complete”, which means 90–99% of the genome is sequenced with no gaps, and that all ORFs are generated (Figure 5).

Following the use of the nanopore sequencing protocol, drop out of amplicons 7, 8 and 9 were observed in the GC-rich region, resulting in no PCR amplicons using this protocol [17]. This bottleneck was resolved by redesigning three pairs of long-read primers for regions 7, 8 and 9, which facilitated the generation of PCR amplicons in this problematic region (Figure 3). The GC-rich region is not conserved between PPRV isolates and poses difficulty for both PCR nucleotide amplification and primer design [74]. Equally, studies have shown that this region has led to the absence of or low nucleotide sequence coverage in the PPRV genome [66]. Next-generation sequencing technologies, such as Illumina and nanopore MinION, generate data of sufficient depth to characterize PPRV strains, but in most cases with no or very low sequence coverage at the GC-rich junction (between nucleotides 4444 and 5526 within the genome) and 3′ and 5′ genome extremities [17]. The missing short sequences are usually amplified using a new set of primers. The genome extremities are also amplified using rapid amplification of cDNA ends by polymerase chain reaction (RACE PCR) [74]. The amplified fragments are later confirmed by Sanger dideoxynucleotide cycle sequencing [69]. Similarly, a systematic drop out of problematic amplicons 18 and 76 was noticed during sequencing of SARS-CoV-2, which has led to the modification of the ARTIC nanopore sequencing protocol [75]. Within this study, the coverage depth was very uneven across the genome (Figure 4). Thus, the accumulation of genetic diversity in PPRV over time may necessitate further changes in the nanopore sequencing protocol. A larger field study is needed to allow the collection of sufficient data to assess the robustness of the protocol. 

The sequence coverage was much higher, up to 8000 reads per position (Figure 4). The majority of the reads mapped against the reference genome with an average of 96.73% (Table 3). This indicated high-quality viral RNA, with no degradation of the viral RNA genomes in selected PPR-positive samples. However, very low reads were observed in the GC-rich region at the genome position between 4 and 6 kb (Figure 4). The GC-rich region was approximately 1080 nucleotides long, with an average of 68.15% GC content residing between the M and F genes of PPRV. Equally, previous studies have described that the M and F untranslated intergenic region is the longest intergenic region in the PPRV genome and is very rich in GC content (66–72%) [9]. Owing to secondary and hairpin structures in the GC-rich region, this region is an extremely difficult PCR target for both primer design and nucleotide amplification [76]. The development of overlapping long-read primers targeting this region was critically important to generate nucleotide sequences covering this region (Figure 4). Similarly, previous studies have confirmed that the GC-rich and genome extremities were regions with the lowest or absent sequence coverage in all four known PPRV lineages [17]. 

Genomic comparison of Tanzanian field isolates, together with other available complete genomes of PPRV available on GenBank, showed a high level of sequence conformity, and Tanzanian isolates clustered together with other isolates of lineage III PPRV (Figure 6). Phylogenetic analysis revealed a high nucleotide identity (96.19–99.24%) with lineage III PPR viruses currently circulating in East Africa, indicating a common origin. The M and H sequences were the most conserved of the genes with lineage III PPR viruses in East Africa (Table 4). Equally, previous studies showed that between isolates, the PPRV genome is relatively conserved, with a maximum divergence of 12% at the nucleotide level and 7% at the amino acid sequence levels [59]. Further analysis of posterior probabilities suggests a strong historical and geographic connection between the Tanzanian field isolates and PPR viruses isolated in East Africa (Figure 7). These analyses show a very strong likelihood >72% that the lineage III viruses currently circulating in East Africa spread from Ethiopia as the most likely origin, although genomic surveillance for PPRV is poor and a lack of full-genome sequencing from other regions likely biases this result (Figure 8). A phylogeographic method estimated the probability of the root location of an ancestral PPRV and individual lineages as being Nigeria for PPRV, as previously reported [59]. The phylogeographic reconstruction with spatial and temporal information of PPRV isolates has enabled an understanding of the historic emergence and dispersal patterns involved in PPRV evolution [29,59]. As the available number of complete genome sequences is very small in East Africa, to explore the relationship between African virus isolates, further sequences and phylogeographic analyses are needed. As with many other areas, partial sequences are most commonly available but do not enable a thorough analysis of PPRV genetics (Table 1). Certainly, the paucity of full genome data significantly limits the opportunity to evaluate genetic changes outside of the target sequences. Certainly, a more thorough genetic analysis is critical in the study of PPRV pathogenesis and viral evolution studies, alongside the development of novel diagnostics and therapeutic tools for PPR control and eradication (10).

Peste des petits ruminants may have passed unrecognized for several years in some areas of Tanzania, because it is often confused with other diseases that cause respiratory problems and mortality in small ruminant populations [28,29]. A retrospective study that was conducted on archived samples collected from Ngorongoro district between 1998 and 2004 demonstrated anti-PPRV-specific antibodies in samples collected in 2004, indicating that PPR was likely present in Tanzania before it was officially confirmed following diagnostic evaluation in 2008 [35]. The presence of small ruminant diseases that can be considered in differential diagnosis, including bluetongue, contagious caprine pleuropneumonia, Orf disease, capripox and foot and mouth disease, often overlap in syndromic evaluation, leaving laboratory diagnosis as the only mechanism to diagnose PPR [28,30,31]. In addition, secondary infection caused by *Pasteurella multocida* and *Mannheimia haemolytica* can also complicate syndromic diagnosis [32]. Molecular confirmation of the occurrence of PPR in PPR-free zones and endemic settings is critically important for the accurate diagnosis and confirmation of outbreaks [33]. 

To enable the successful eradication of PPR, rapid diagnosis remains a cornerstone for the implementation of control measures, including vaccination, quarantine and possible stamping out [36,77]. The wide host susceptibility of species to PPR presents logistic challenges around sampling, diagnosis and diagnostic protocols need to be adequately validated for atypical species affected, and the type of sample being collected [78,79,80]. The application of Oxford nanopore MinION sequencing technology demonstrated it to be an effective and rapid option for the molecular sequencing of PPRV. Importantly, the sequencing protocol described has been developed and implemented for the sequencing of PPRV in the Molecular Virology Laboratory at Sokoine University of Agriculture in Tanzania as part of this study. This enhanced capability in an endemic setting has demonstrated that Oxford nanopore MinION sequencing is a viable option for minimally equipped diagnostic facilities in low-income and resource-constrained countries. Going forward, the Oxford nanopore MinION sequencer is likely to be an important tool in providing rapid and in-depth genomic information of circulating PPRV strains during the eradication programme. Critically, it will play a significant role in defining PPR outbreaks by enabling the detection of cryptic foci, as well as demonstrating virus circulation in areas where inadequate vaccine deployment may have occurred. 

## 5. Conclusions

Two complete genomes of lineage III PPRV from Tanzania field isolates were generated for the first-time using Oxford nanopore MinION, in our Molecular Virology Laboratory at the Sokoine University of Agriculture in Tanzania. The development of this protocol in a resource-limited setting has demonstrated its utility for PPRV and other viral pathogens where full-genome sequence data acquisition is beneficial. Phylogenetic analysis of the complete genomes revealed a high nucleotide identity with lineage III PPR viruses currently circulating in neighbouring countries (Kenya, Burundi and Uganda), indicating a common origin. There is a very strong likelihood, >72%, that the lineage III viruses currently circulating in East Africa spread from the Ethiopia 1994 outbreak as the most likely origin. However, the coverage depth was uneven across the genome, with amplicon dropout at the GC-rich region and genome termini. A larger field study is required to enable the collection of sufficient data to assess the robustness of the nanopore sequencing technology and to validate the protocol. 

## Figures and Tables

**Table 1:** Molecular epidemiological studies of peste des petits ruminants in different districts of Tanzania, based on partial N gene and complete genome sequence analysis from 2010 to 2020. The sequences from these studies clustered into three lineages (II, III and IV) and they were isolated from domestic sheep and goats, and a wild small ruminant (Grant’s gazelle).

Region/District	Study Period	Host	Sequence	Lineage	References
Arusha, Kilimanjaro, Manyara and Tanga	2010	sheep and goats	partial	III	[40]
Tandahimba and Newala	2011	sheep and goats	-	-	[41]
Mvomero	2013	sheep and goats	-	-	[42]
Ngorongoro and Mvomero	2013	goats	partial	III	[43]
Ngorongoro	2014	sheep and Grant’s gazelle	partial	II	[44]
Tandahimba	2015	sheep and goats	partial	II and IV	[45]
Ngorongoro	2015	sheep and goats	partial	III	[46]
Ngorongoro	2016	sheep and goats	-	-	[47]
Mbeya, Iringa, Dodoma Morogoro, Pwani Serengeti, Tanga and Arusha	2018	sheep and goats	-	-	[48]
Mvomero	2020	sheep and goats	complete	III	[17]

**Table 2:** List of long-read primers, 7, 8 and 9, that were developed to amplify an extremely difficult PCR target, the GC-rich region between matrix (M) and fusion (F) protein genes of PPRV.

Primer Name	Fragments	Sequence (5′ to 3′)	Tm *	GC%	Position on Genome		Fragment Size (bp)
					Start	End	
PPRV7LK	F7	CAACAACACTCCGCTGTCCT	60.30	55.00	3778	3796	770
PPRV7RK		GAGTGGCTGTGTTGGTGCT	60.53	57.89	4548	4530	
PPRV8LK	F8	CAAGCCGTCCTACAGCCATC	60.81	60.00	4353	4372	895
PPRV8RK		GTCCTCCCTCGGTCTGTCT	60.00	63.16	5248	5229	
PPRV9LK	F9	GAGGACACCCAACCACCGAAAC	59.00	59.09	4972	4993	815
PPRV9RK		ACAGAGCATCCTCTACAGGCTT	55.00	50.00	5787	5766	

* Tm; melting temperature, GC%; GC content.

**Table 3:** Results from full-genome sequencing of PPRV field isolates from goats in Ngorongoro and Momba districts of the northern and southern highland of Tanzania, using Oxford Nanopore MinION sequencer.

Sample	Raw Reads	Total bp	N50 Length (bp)	Reads Mapped to PPRV	Average Coverage Reads	Genome Coverage >50× (%)	Genome Coverage >25× (%) Source	PPRV Lineage
Ngorongoro	1,881,426	2,203,973,564	793	1,784,633	4575	99.2	99.8	III
Momba	1,712,393	1,605,543,198	816	1,688,419	3906	98.7	99.5	III

**Table 4:** Publicly available complete genome sequences of peste des petits ruminants virus strains from East Africa and selected strains from other parts of Africa and Asia used for comparative genomic analysis in this study.

Isolate Name	GenBank	Country of Origin	Year of Collection	Lineage	Percentage Nucleotide Identity with Tanzania /2016 PPRV Isolate	Percentage Nucleotide Identity with Tanzania/ 2018 PPRV Isolate	Host Species	Reference
Tanzania/2016	MW960272	Tanzania	2016	III	100.00	97.39	goat	This study
Tanzania/2018	MZ322753	Tanzania	2018	III	97.39	100.00	goat	This study
KN5/2011	KM463083.1	Kenya	2011	III	99.24	97.92	goat	[60]
B3	MK686066.1	Burundi	2017	III	98.44	97.22	goat	[61]
Uganda 2012	KJ867543.1	Uganda	2012	III	97.38	96.19	goat	[62]
Ethiopia 1994	KJ867540.1	Ethiopia	1994	III	95.55	95.57	goat	[62]
UAE 1986	KJ867545.1	United Arab Emirates	1986	III	94.49	94.57	Dorcas gazelle	[62]
Oman 1983	KJ867544.1	Oman	1983	III	94.47	94.56	goat	[62]
Nigeria/75/1	HQ197753.1	Nigeria	1976	II	88.58	88.73	goat	[63]
Benin/B1/1969	KR781450.1	Benin	1969	II	88.85	89.00	goat	[64]
Ng76/1	EU267274.1	Nigeria	1976	II	88.50	88.66	goat	[65]
Ghana/2010	KJ466104.1	Ghana	2010	II	87.69	87.81	sheep	[66]
Benin/10/2011	KR781449.1	Benin	2011	II	87.59	87.72	sheep	[64]
ICV89	EU267273.1	Cote d’Ivoire	1989	I	87.92	88.02	goat	[65]
Ethiopia 2010	KJ867541.1	Ethiopia	2010	IV	87.20	87.35	goat	[67]
Georgia/2016	MF737202.1	Georgia	2016	IV	86.94	87.04	sheep	[68]
Morocco 2008	KC594074.1	Morocco	2008	IV	87.35	87.44	goat	[28]
Turkey/2018	MN657232.1	Turkey	2018	IV	86.85	87.01	sheep	[69]
Mongolia/2016	KY888168.1	Mongolia	2016	IV	86.86	86.88	sheep	[70]
China/33/2007	KX421388.1	China	2007	IV	87.30	87.36	goat	[71]
China/Tibet/07	FJ905304.1	China	2007	IV	87.28	87.34	goat	[72]

## Data Availability

The nanopore datasets generated during this research are publicly available at the NCBI GenBank (accession numbers: MW960272 and MZ322753).
